# Case report: A case of anti-recoverin antibody-positive encephalitis exhibiting Cotard and Capgras delusions that was successfully treated with electroconvulsive therapy

**DOI:** 10.3389/fpsyt.2024.1330745

**Published:** 2024-01-25

**Authors:** Takaki Akahane, Naomi Takahashi, Ryota Kobayashi, Konoka Nomura, Masakazu Akiho, Yukihiro Shikama, Keisuke Noto, Akihito Suzuki

**Affiliations:** ^1^ Department of Psychiatry, Okitama Public General Hospital, Yamagata, Japan; ^2^ Department of Neurology, Okitama Public General Hospital, Yamagata, Japan; ^3^ Department of Psychiatry, Yamagata University School of Medicine, Yamagata, Japan; ^4^ Department of Radiology, Okitama Public General Hospital, Yamagata, Japan

**Keywords:** anti-recoverin antibody, encephalitis, ECT, case report, Cotard delusion, Capgras delusion, rCBF

## Abstract

Recoverin is a neuron-specific calcium-binding protein that is mainly located in the retina and pineal gland. Few reports have described patients with anti-recoverin antibody-positive encephalitis, and no cases of psychosis associated with this encephalitis have been reported. We report a patient with anti-recoverin antibody-positive encephalitis with Cotard and Capgras delusions who was successfully treated with electroconvulsive therapy (ECT). The patient was a 25-year-old woman. She exhibited disorientation, executive function deficits, tremors in the upper limbs, generalized athetoid-like involuntary movements, hallucinations, incontinence, and fever, which led to her admission to our hospital. Upon admission, she complained of Cotard delusions. Various diagnostic tests, including cerebrospinal fluid analysis, antibody screening, and brain imaging, were unremarkable, except for positivity for serum anti-recoverin antibodies, non-specific general slowing on electroencephalography and decreased regional cerebral blood flow (rCBF) in the frontal and occipital lobes, and increased rCBF in the basal ganglia and pons on single-photon emission computed tomography. She was eventually diagnosed with encephalitis positive for anti-recoverin antibodies and treated with immunoglobulins and steroids. Her neurological symptoms improved temporarily, but three months later, psychiatric symptoms, i.e., suicidal thoughts and Cotard and Capgras delusions, were exaggerated. After ECT, her condition significantly improved. In conclusion, the present report suggests that pineal gland dysfunction due to anti-recoverin antibody or its cross-reactivity with neuron-specific calcium-binding proteins may contribute to the neuropsychiatric symptoms observed in anti-recoverin antibody-positive encephalitis and that ECT can be a viable treatment option if immunotherapy proves ineffective. Additionally, decreased rCBF in the prefrontal cortex may be associated with the clinical features of Capgras and Cotard delusions.

## 1 Introduction

Autoimmune encephalitis is a form of autoimmune-mediated disease affecting the central nervous system. Clinical features of autoimmune encephalitis include various neuropsychiatric symptoms, including altered consciousness, seizures, memory deficits, mood changes, delusions, hallucinations, and catatonia (1, 2). Although the specific brain regions or neural circuits associated with these psychiatric symptoms remain unclear, abnormalities in the right hemisphere, insular cortex, prefrontal cortex, frontoparietal circuits, and midline structures have been implicated in certain types of delusions, particularly Cotard and Capgras delusions (3, 4). Autoimmune encephalitis can be triggered by antibodies against neuronal cell-surface antigens, such as the N-methyl-D-aspartate (NMDA) receptor, leucine-rich glioma-inactivated 1 (LGI1), contactin-associated protein-like 2 (Caspr2), alpha-amino-3-hydroxy-5-methyl-4-isoxazolepropionic acid (AMPA) receptor, gamma-aminobutyric acid-B (GABA-B) receptor, and dipeptidyl-peptidase-like protein 6 (DPPX), as well as antibodies against intracellular antigens, such as Hu, Ma2, and glutamic acid decarboxylase (1).

Recoverin is a neuron-specific calcium-binding protein that is mainly located in the retina and pineal gland (5). Antibodies against recoverin are found in the serum of patients with cancer-associated retinopathy (6). There have been few reports describing patients with anti-recoverin antibody-positive encephalitis exhibiting neuropsychiatric symptoms such as ataxia, seizures, altered consciousness, agitation, and depressive mood (7–12). Notably, there have been no cases of psychosis associated with this encephalitis. Multiple abnormal examination findings have been reported in relation to this condition. These include pleocytosis observed in cerebrospinal fluid tests, leukoaraiosis and hyperintense regions at the basal ganglia as observed in magnetic resonance imaging, reduced radiotracer uptake at the basal ganglia as seen in dopamine transporter single emission computed tomography, and generalized slow waves observed in electroencephalography. It is worth noting that the results have been inconsistent across different studies (7–12). Regarding treatment, immunotherapies involving steroids, immunoglobulins, and rituximab have shown promising results in improving symptoms in certain cases (8, 10, 11). Here, we report the case of a patient with anti-recoverin antibody-positive encephalitis exhibiting Cotard and Capgras delusions, who was successfully treated with electroconvulsive therapy (ECT).

## 2 Case report

The patient was a 25-year-old woman with no significant medical or family history. The patient provided written informed consent to report her clinical course when she was in a state of clear consciousness after being discharged from our hospital. This report was approved by the Ethical Review Committee of Yamagata University Faculty of Medicine.

Nine days before admission to our hospital, she displayed a lack of response to her mother’s calls, impaired executive function, such as an inability to open and close a pencil case, disorientation, tremors in the upper limbs, visual hallucinations of suicide victims, incontinence, and wandering. Seven days prior to admission, she developed a slight fever and was admitted to our hospital.

**Figure 1:**
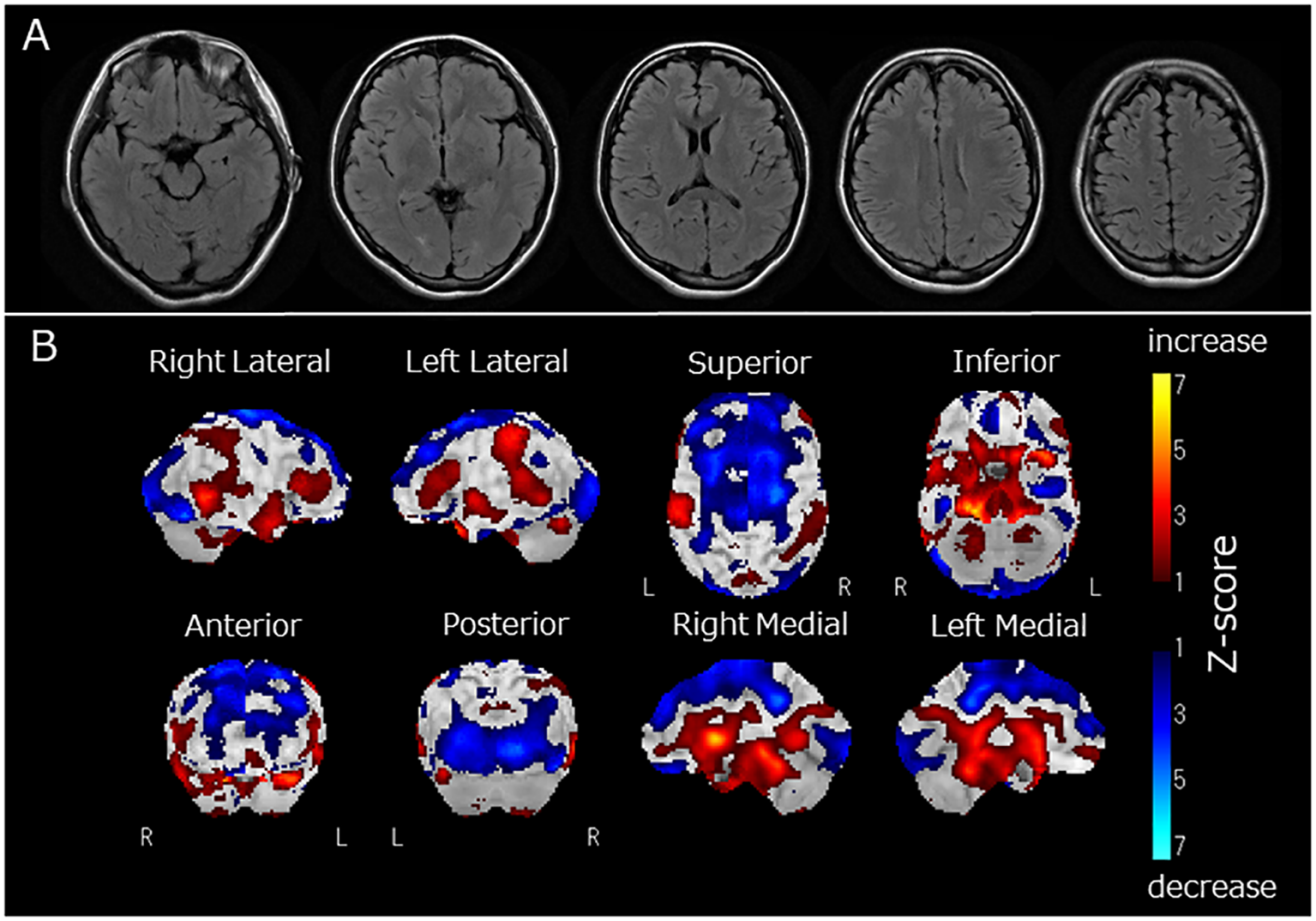
**(A)** Fluid Attenuated Inversion Recovery magnetic resonance imaging of the brain, **(B)** regional cerebral blood flow (rCBF) using single-photon emission computed tomography (SPECT) with ^123^I-iodoamphetamine. Fluid-attenuated inversion recovery magnetic resonance imaging revealed no apparent brain atrophy or high-intensity areas **(A)**. SPECT using a ^123^I-iodoamphetamine was performed, and SPECT data were analyzed by 3D stereotactic surface projections (3D-SSP) included in “medi+FALCON version 1.4” (Nihon Medi-Physics Co. Ltd., Tokyo, Japan). Z-score maps of regional cerebral blood flow (rCBF) were created based using 3D-SSP. The SPECT images showed decreased rCBF in the occipital and frontal lobes **(B)**. In contrast, rCBF increased in the basal ganglia and pons **(B)**.

Upon admission, the patient exhibited agitation, restlessness, incoherent thoughts, disorientation, and poor conversational fluency. She also displayed generalized athetoid-like involuntary movements and weakness in the lower limbs. She showed a fever of 39.5 °C and an increased heart rate of 156/min. Tendon reflexes and pupillary responses were unremarkable, and no nuchal rigidity or pathological reflexes were observed. She complained of Cotard delusions such as “I am dead,” “I have no uterus,” and “my heart is not beating.” Her laboratory blood tests showed increased levels of white blood cells at 18,000/μL, total bilirubin at 1.70 mg/dL, lactate dehydrogenase at 410 U/L, and creatine kinase at 1,705 U/L. C-reactive protein levels were measured at 0.01 mg/dL. Thyroid function (free T3, free T4, and thyroid stimulating hormone levels) was normal. A toxicological screening test and blood psychotropic level measurements were not performed because her parents, who lived with her, claimed that she did not consume alcohol, take prescribed psychotropics, or use illegal drugs. She underwent a lumbar puncture, and the results showed an opening pressure of 15 cm H_2_O, no pleocytosis with 2 counts/μL, and a normal IgG index of 0.43, along with normal protein and glucose levels. PCR results for herpes simplex virus DNA in the cerebrospinal fluid was negative. There were no IgG or IgM antibodies found against herpes simplex virus, varicella-zoster virus, and cytomegalovirus in the cerebrospinal fluid. The screening for antibodies against the NMDA receptor, LGI1, Caspr2, AMPA receptor, GABA-B receptor, and DPPX in the cerebrospinal fluid all yielded negative results. Serum antibodies against nuclear, NH_2_ terminal of alpha-enolase (NAE), thyroglobulin, and thyroid peroxidase were also negative. However, she tested slightly positive for serum anti-recoverin antibodies (BML Inc., Tokyo, Japan). The contrast-enhanced magnetic resonance imaging of the head was unremarkable (**Figure 1A**). Her electroencephalogram showed diffuse nonspecific general slowing without any signs of epileptic discharge. Other examinations, such as whole-body computed tomography and magnetic resonance imaging of the spine, pelvic region, and ovaries, did not reveal any remarkable abnormalities, including tumors. Tumor markers, including CEA, AFP, CA125, CA19-9, SLX, SCC antigen, SYFRA21-1, ProGRP, NSE, CA15-3, sIL-2L, and BCA225, all yielded negative results. An ophthalmological examination revealed no signs of retinopathy.

She was suspected of having autoimmune encephalitis and was treated with two cycles of intravenous immunoglobulins 20,000 mg/day for 5 days, and three cycles of steroid pulse therapy using methylprednisolone 1,000 mg/day for 3 days. Levetiracetam 1,000 mg/day and valproate (800 mg/day) were administered to prevent epileptic seizures. Approximately one month after admission, her neurological symptoms, such as disorientation and poor conversational fluency, improved. The dose of prednisolone was tapered to 17.5 mg/day. For psychiatric symptoms, olanzapine (up to 20 mg/day), risperidone (up to 6 mg/day), lorazepam (up to 6 mg/day), quazepam (20 mg/day), and lemborexant (5 mg/day) were used, but these treatments were discontinued due to a lack of apparent improvement.

Two months after admission, she displayed pessimistic thoughts, suicidal ideation, Cotard delusions, and Capgras delusions such as “Other persons, who have the same facial features as my parents but who have been replaced, live in my home. My real parents are dead.” She attempted suicide by jumping or hanging because she believed that her entire family was dead and that there was no reason to live. Regional cerebral blood flow (rCBF) was evaluated using single-photon emission computed tomography (SPECT) with ^123^I-iodoamphetamine. rCBF was decreased in the bilateral frontal and occipital lobes and increased in the bilateral basal ganglia and pons (**Figure 1B**). Fourteen sessions of bilateral ECT were conducted. The decision to employ ECT was based on the urgent need to address frequent suicidal attempts. ECT has been recognized for its efficacy in preventing such attempts (13). Additionally, it has proven to be effective in treating autoimmune encephalitis (2). Two months and three months after admission, two serum examinations were conducted and both showed a strong positive result for anti-recoverin antibodies. After ECT, her psychiatric symptoms, including Cotard delusions, Capgras delusions, and suicidal thoughts, markedly improved. At 8 months, steroid therapy was discontinued, and the patient was discharged from the hospital after physical and verbal rehabilitation. One year after discharge, the patient remained in remission.

## 3 Discussion

In the present case, there was an acute onset and rapid progression within 9 days of neuropsychiatric symptoms, which included delusions, hallucinations, altered consciousness, and focal neurological findings such as tremors, athetoid-like involuntary movements, weakness, and impaired executive function (**Table 1**). The patient tested positive for serum anti-recoverin antibodies. Diffuse, nonspecific general slowing was observed on electroencephalography, along with decreased rCBF in the frontal and occipital lobes and increased rCBF in the basal ganglia and pons on SPECT. Treatment with immunotherapy and ECT led to the remission of the patient’s neuropsychiatric symptoms. Viral and other autoimmune encephalitides were ruled out, as there was no detection of herpes simplex virus DNA or various antibodies associated with autoimmune encephalitis (1). While Cotard and Capgras delusions have been rarely reported in patients with schizophrenia (14), the diagnosis of schizophrenia was considered unlikely in this case due to the presence of autonomic symptoms such as fever and increased heart rate, neurological symptoms, and abnormal findings on electroencephalography. Additionally, complete remission persisted for 1 year after discontinuation of antipsychotic treatment. Therefore, it was considered that the present case had possible autoimmune encephalitis caused by anti-recoverin antibodies, according to the diagnostic criteria proposed by Graus et al. (1).

**Table 1:** Characteristics, clinical symptoms, examination findings, and treatments in the patient with anti-recoverin antibody-positive encephalitis.

Age	25 years old
Sex	Female
Retinopathy	−
Malignancy	−
Neuropsychiatric symptoms	Cotard delusion, Capgras delusion, hallucinations, suicidal thought, altered consciousness, tremors, athetoid-like involuntary movements, weakness, impaired executive function
Anti-recoverin antibody	+
Other antibodies	−
Brain imaging	Unremarkable MRI findings, decreased rCBF in the frontal and occipital lobes and increased rCBF in the basal ganglia and pons on SPECT.
EEG	Diffuse, nonspecific general slowing
CSF abnormalities	−
Treatment	Steroid, immunoglobulin, and ECT
Outcome	Improved

ECT, electroconvulsive therapy; MRI, magnetic resonance imaging; rCBF, regional cerebral blood flow; SPECT, single-photon emission computed tomography.

The effects of anti-recoverin antibodies on the central nervous system are not fully understood. Recoverin is expressed in the pineal gland (6) as well as the retina. The pineal gland, which secretes melatonin, is involved in the modulation of circadian rhythms, regulation of sleep, reproductive physiology, and immunological regulation (15). It has been shown that patients with psychosis and mood disorders have a smaller pineal gland volume, lower blood levels of melatonin, and aberrant patterns of melatonin secretion (15), whereas the administration of melatonin to hospitalized patients is related to a reduction in delirium incidence (16). These studies suggest that pineal gland function is involved in psychosis, mood disorders, and delirium. Thus, it is possible that altered dysfunction of the pineal gland caused by anti-recoverin antibodies may have been associated with the neuropsychiatric symptoms observed in this case. Meanwhile, recoverin has 40–55% sequence identity with other neuron-specific calcium-binding proteins, i.e., hippocalcin, neuronal calcium sensor-1, and visinin-like protein (17). These neuron-specific calcium-binding proteins are widely expressed in the brain, including the neocortex, caudate, brain stem, hippocampus, amygdala, putamen, forebrain, and cerebellum (5), and are reportedly associated with various neuropsychiatric diseases, such as intellectual disability, autism, attention deficit disorder, dystonia, and Alzheimer disease (18). Therefore, as suggested by Kitazaki et al. (11), the cross-reactivity of the anti-recoverin antibody with these neuron-specific calcium-binding proteins may induce autoimmune encephalitis.

ECT is effective in treating anti-NMDA receptor encephalitis, particularly in patients with catatonia (2), presumably because of its effects on glutamate, GABA, serotonin, and dopamine neurotransmission (19). In this case, immunotherapy (immunoglobulins or steroids) and antipsychotics were insufficient to manage intense and severe psychiatric symptoms such as Cotard delusions, Capgras delusions, and suicidal ideation, whereas ECT treatment yielded excellent outcomes for these psychiatric symptoms. Previous reports on anti-recoverin antibody-positive encephalitis have shown that immunotherapy improved symptoms in some cases (8, 10, 11), but not in others (9, 10). Thus, the present case suggests that ECT may be the next treatment option when immunotherapy is ineffective for this type of encephalitis.

In this case, we observed Cotard and Capgras delusions. Cotard and Capgras delusions belong to delusional misidentification syndrome, caused by various organic brain diseases such as dementia, cerebrovascular disease, and encephalitis, as well as psychiatric disorders such as schizophrenia and depression (20, 21). These delusions are reportedly developed by two factors (20): abnormal perception leading to sensations of derealization or depersonalization, which is associated with dysfunction of the insular cortex, and abnormal rationalization towards external causes (Capgras delusion) and internal causes (Cotard delusion), which are related to dysfunction of the prefrontal cortex (3). The present case displayed decreased rCBF in the frontal and occipital lobes and increased rCBF in the basal ganglia and pons. Therefore, dysfunction in the prefrontal cortex, reflected as decreased rCBF, might be associated with the clinical features of Capgras and Cotard delusions observed in this case.

One limitation of the report is that the pathological significance of anti-recoverin is not well characterized. Consequently, the possibility that other undetected antibodies might be responsible for the neuropsychiatric symptoms in this case and that anti-recoverin antibodies co-existed by chance, cannot be ruled out. Secondly, this report presents a single case, highlighting the need to accumulate more cases in order to draw conclusive findings for clinical practice in anti-recoverin antibody-positive encephalitis.

In conclusion, the present case report suggests the importance for clinicians to assess the presence of anti-recoverin antibodies and to consider this encephalitis in the differential diagnosis when confronted with acute onset neuropsychiatric symptoms, including Cotard and Capgras delusions. In cases where immunotherapy is not effective, ECT may be the next treatment option for this encephalitis. Altered dysfunction of the pineal gland caused by anti-recoverin antibodies or cross-reactivity of anti-recoverin antibodies with neuron-specific calcium-binding proteins may be associated with neuropsychiatric symptoms observed in anti-recoverin antibody-positive encephalitis. This report also suggests that decreased rCBF in the prefrontal cortex may be associated with the clinical features of Capgras and Cotard delusions. It is important to gather more cases to confirm these findings.

## Data availability statement

The original contributions presented in the study are included in the article/supplementary material. Further inquiries can be directed to the corresponding author.

## Ethics statement

The studies involving humans were approved by The Ethical Review Committee of Yamagata University Faculty of Medicine. The studies were conducted in accordance with the local legislation and institutional requirements. The participants provided their written informed consent to participate in this study. Written informed consent was obtained from the individual(s) for the publication of any potentially identifiable images or data included in this article.

## Author contributions

TA: Conceptualization, Investigation, Writing – original draft. NT: Conceptualization, Investigation, Writing – original draft. RK: Data curation, Formal Analysis, Investigation, Methodology, Visualization, Writing – review & editing. KoN: Conceptualization, Data curation, Investigation, Writing – original draft. MA: Data curation, Formal Analysis, Investigation, Methodology, Writing – review & editing. YS: Conceptualization, Investigation, Supervision, Writing – review & editing. KeN: Data curation, Formal Analysis, Writing – review & editing. AS: Conceptualization, Formal Analysis, Investigation, Methodology, Supervision, Writing – original draft.
